# The ethical application of biometric facial recognition technology

**DOI:** 10.1007/s00146-021-01199-9

**Published:** 2021-04-13

**Authors:** Marcus Smith, Seumas Miller

**Affiliations:** 1 Centre for Law and Justice, Charles Sturt University, Canberra, Australia; 2 Australian Graduate School of Policing and Security, Charles Sturt University, Canberra, Australia; 3 Delft University of Technology, The Hague, The Netherlands; 4 University of Oxford, Oxford, UK

**Keywords:** Biometric technologies, Biometric facial recognition, Surveillance, Privacy, Security

## Abstract

Biometric facial recognition is an artificial intelligence technology involving the automated comparison of facial features, used by law enforcement to identify unknown suspects from photographs and closed circuit television. Its capability is expanding rapidly in association with artificial intelligence and has great potential to solve crime. However, it also carries significant privacy and other ethical implications that require law and regulation. This article examines the rise of biometric facial recognition, current applications and legal developments, and conducts an ethical analysis of the issues that arise. Ethical principles are applied to mediate the potential conflicts in relation to this information technology that arise between security, on the one hand, and individual privacy and autonomy, and democratic accountability, on the other. These can be used to support appropriate law and regulation for the technology as it continues to develop.

## Introduction

Biometric facial recognition is one of the most significant and rapidly developing artificial intelligence (AI) technologies currently available for security and law enforcement purposes. In countries such as the United Kingdom, United States and Australia, the use of this technology and its application has been widely debated. In early 2020, it became publicly known that governments were using information technology developed by a private technology company to search billions of social media images to identify suspects (Hill 2020). In authoritarian states such as China, a social credit system has been established, facilitated by an extensive biometric surveillance network, illustrating the potential endpoint for liberal democracies if they continue on the current trajectory of technology adoption.

The first part of the article considers the rise of this technology, introducing a number of associated issues. The second part outlines current applications and legal developments, incorporating significant examples in this area in Australia, the United States and the United Kingdom. These jurisdictions were selected because they have been more proactive in their adoption of the technology than Europe, which has banned and/or highly regulated its use (Stupp 2020). The third part of the article proceeds to undertake an ethical analysis of the use of biometric facial recognition, drawing on these developments. Potential conflicts between security, on the one hand, and individual privacy and autonomy, and democratic accountability, on the other, are analysed. Security and public safety are fundamental values in liberal democracies; however, these countries are also committed to individual privacy, autonomy, democracy and democratic accountability, and these fundamental principles must continue to be valued in liberal democracies, notwithstanding the benefits to security that biometric facial recognition technology can provide.

## The rise of biometric facial recognition

Biometric facial recognition is a form of AI that involves the automated extraction, digitisation and comparison of the spatial and geometric distribution of facial features to identify individuals. Using a digital photograph of a subject’s face, a contour map of the position of facial features is converted into a digital template, using an algorithm to compare an image of a face with one stored in a database. Images can be collected from repositories of passport or drivers licence photographs, or from the vast number of images that have been uploaded to social media sites and the internet. Biometric facial recognition systems can be integrated with the closed circuit television systems that already exist in public and private spaces to identify people in real time (Smith et al. 2018). Biometric technologies are part of a shift taking place in society towards automated decision-making processes that involve limited human intervention. While current literature on the subject notes that the ‘displacement of agency from humans to machines, raises ethical questions about mediated social sorting and discrimination’ (Marciano 2019; 134), it is more often focused on sociological analysis of the issues and there is a gap in applied ethical analysis that can provide a foundation for law and policy solutions.

The expanding use of this technology raises a number of pressing ethical concerns for liberal democracies (Kleinig et al. 2011). The concerns associated with biometric facial recognition arise in large part from potential conflicts between the pursuit of ethical values and their application in various domains. The values in question are constitutive of liberal democracy, and include security, individual privacy and autonomy, and democratic accountability. These domains include border security, criminal investigation, national security and private sector commercialisation of data. Central to the ethical, legal and policy issues is the tension that exists between the legitimate collection of biometric information for law enforcement, national security and government service provision, on the one hand; and the rights to privacy and autonomy in liberal democracies on the other. In Australia, the United States, United Kingdom and other liberal democratic countries, the threat from terrorism over the past 20 years has prompted a number of significant changes to legislation and practices of law enforcement and security agencies. As a result, government agencies today have much greater powers to collect evidence and conduct surveillance, and to do so more proactively, to detect, disrupt and arrest challenging non-state threats like terrorism and transnational crime (Walsh and Miller 2016). The impact of these changes has led to debate regarding whether this more proactive collection of data, such as biometric information, from citizens who have not committed a crime is acceptable, and on the ethics of biometric information collection programs more generally (Henschke 2017).

New approaches to consent and data security are needed to address the rapid expansion in the types of data available and the ways in which it is being used (Kaye et al. 2015). Greater volumes of data are generated and used in novel ways, and instances of large scale data breaches involving institutions, governments and businesses become more common (ANU 2019).^1^ The capacity to integrate biometric and other data (for example, smart phone metadata, financial, medical and tax records) adds to these concerns. Biometric facial image templates can be used in conjunction with digital images sourced from closed circuit television (CCTV), phone metadata, and internet history, to provide an increasingly complete picture of an individual’s movements and lifestyle.

Developments in authoritarian states provide further insights into the potential impact of the use of biometrics in the absence of democratic accountability. China utilises biometric facial recognition systems to identify individuals in public places via CCTV who are suspected of minor crimes, such as jaywalking (Qiang 2019) or for shaming citizens engaging in ‘uncivilised behaviour’ such as ‘wearing pyjamas in public’ (BBC 2020a). China’s social credit system rewards and punishes citizens on the basis of social norm compliance or non-compliance, honesty and courtesy, in concert with other data analysis capabilities that facilitate tracking, such as global positioning system data, internet use, and financial transaction history. The implications of a low social credit score for Chinese citizens include travel bans, and exclusion from private schools and higher status professions. It is believed that more extensive surveillance and discrimination using facial recognition and other biometrics is being used in relation to ethnic minorities such as the Uighurs (Wee and Mozur 2019).

## Current applications and legal developments

There have been significant applications and legal developments in relation to biometric facial recognition in Australia, the United States and the United Kingdom over the past 10 years, and systems continue to advance rapidly. Their use in association with passports at international airports has been well established for more than a decade and they continue to play an important role in border control systems. In recent years, this technology has become increasingly important for law enforcement investigations (ACIC 2019). Legislation to facilitate the integration of facial images from passports and drivers licences into a national database for use by law enforcement and other government agencies is being introduced in some countries, while in others, similar databases are likely being introduced without the public’s knowledge (PJCIS 2019). As discussed below, over the past year there have been some significant developments: social media images have become integrated into biometric facial recognition systems; and the technology has also been subject to judicial review in courts.

### Australia

Developments in Australia illustrate the increasing take up of the technology over the past decade. In 2009, biometric facial recognition compatibility was introduced in the state of New South Wales (NSW) through an amendment to the regulations governing drivers’ licences, allowing these images to be searched using biometric systems.^2^ In 2015, a regulation was introduced, permitting the release of biometric drivers licence photographs to NSW Police, as well as the Australian Federal Police and the Australian Security Intelligence Organisation.^3^ Photographs can be released for biometric matching for the purposes of investigation of ‘relevant criminal activity’,^4^ a ‘terrorist act’, the ‘threat of a terrorist act’,^5^ without warrant or the knowledge or consent of individuals concerned. This change to the regulations, as opposed to legislation, occurred without public debate or knowledge that biometric facial recognition capabilities had been implemented.

Also in 2015, the Federal Government announced that a national facial biometric matching Capability was being developed and would enable state and federal agencies to share facial templates for the purpose of biometric facial identification (Keenan 2015). It was to have the capacity for verifying identity through one-to-one matching of documents and one-to-many searching of databases, to identify unknown persons. In additional to state and territory drivers licence photographs, it was also to include all passport images (Australian Government 2017). The government initially sought to implement the system by amending state and Commonwealth regulations. Following public criticism that they were trying to introduce the database without having it subjected to proper scrutiny, legislation was introduced in the national parliament to provide the legal authority for the database (Mann and Smith 2017).

The subsequent Identity-matching Services Bill 2019 and Australian Passports Amendment (Identity-matching Services) Bill 2019 was debated in parliament but was not enacted into law, following recommendations of an inquiry by the Parliamentary Joint Committee on Intelligence and Security. The bills sought to authorise the Department of Home Affairs to develop, operate and maintain: an ‘interoperability hub’ through which participating agencies and organisations can request and transmit biometric facial images and information contained in government identity documents such as driver licenses. Submitters to the inquiry, and ultimately the committee itself, determined that there were insufficient oversight mechanisms included in the legislation for a system with such significant capabilities. This included who would be authorised to access the database and under what circumstances, such as whether warrants and threshold offences would be required. Another point that was highlighted was the proposal that the private sector has limited access to the database to verify the identity of those they did business with, creating further regulatory complexities and risks (Petrie 2019). Amended legislation to establish the database is expected to be reviewed by the Australian Parliament in 2020.

### United States

In January 2020, it became widely known that law enforcement agencies in the United States were using a biometric facial recognition algorithm, developed by the company Clearview AI, to search images on the internet to identify suspects (Hill 2020). It has also been reported that police in the United Kingdom and Australia have used Clearview AI’s technology (Bogle 2020). National databases of passport and drivers licence images are relatively small in comparison with internet based technology that has the capacity to search the more than three billion facial images obtained from photographs or video on social media sites and other online sources to identify a suspect (Hill 2020). It has also been reported that Clearview AI not only provides facial recognition software for law enforcement agencies, but may also service private companies in the United States, including Walmart, AT&T, the NBA, Bank of America and Best Buy for their private security purposes.^6^

The Clearview AI application integrates three billion facial images ‘scraped’ from social media which it integrates with a facial recognition algorithm to identify unknown individuals from video or photographs (Hill 2020). This vastly increases the scope of biometric facial recognition, even in comparison with the scale of national databases, which would encompass tens or possibly hundreds of millions of people. Following publication of Clearview AI’s services, the State of New Jersey and social media companies, including Twitter and Facebook, sent cease-and-desist letters asserting that the company was breaching their policies on the use of images (BBC 2020b).

Legal action against Clearview AI has also been taken by a number of parties. In 2020, a class action was commenced against Clearview AI by the law firm Haeggquist and Eck, LLP. The statement of claim alleges that Clearview AI had violated the *California Consumer Privacy Act of 2018* (CCPA) and the *Illinois Biometric Information Privacy Act* (BIPA). The action on behalf of the plaintiffs states that:

- the individuals did not consent to the use or redistribution of photographs, biometric information and identifiers;
- clearview AI ‘scraped’ the images from internet-based websites, in violation of several of the websites’ terms of use;
- clearview AI applied facial recognition software in violation of the CCPA and BIPA;
- clearview AI sold access to photographs, biometric information and identifiers to third-party entities for commercial gain without consent; and
- damages were suffered in terms of the diminution in value of individuals’ biometric information, and identifiers and placed them at risk of privacy violation.^7^

This recent developments add further complexity to the legal and ethical issues associated with biometric facial recognition, with the reported use of the technology by private sector companies such as banks and retailers of particular concern. While legal constraints associated with Clearview AI’s use of images held by social media companies may ultimately threaten its feasibility and ability to provide its services to the private sector; this would not be an issue for a law enforcement agency.

### United Kingdom

In 2019, the High Court of England and Wales considered the use of biometric facial recognition by police to identify suspects in the case *R (on the application of Edward Bridges) v The Chief Constable of South Wales.*^8^ The case concerned the use of AFR Locate^9^ by South Wales Police (SWP). This system applied biometric facial recognition technology to live images, acquired via a camera attached to a van, and compared these to images of persons on a watch list. Mr Bridges, claimed that SWP had processed his image in two locations using the system, and that he was not on any watch list. He argued that this breached his rights under Article 8(1) of the European Convention on Human Rights (ECHR), his ‘right to respect for his private and family life, his home and his correspondence’ and that this was not justified under Article 8(2), as it was not ‘in accordance with the law’ nor ‘necessary in a democratic society’ for any of the relevant purposes under that article, which include public safety and crime prevention.^10^

The court accepted that the use of AFR Locate interfered with Mr Bridges’ privacy rights, but that this was outweighed by the powers of the police to prevent and detect crime. It distinguished biometric facial recognition from other actions of police that require a warrant, because it is less invasive:

A warrant is required to allow the police to enter someone’s private property since otherwise, the act of entering someone's private property without permission would amount to a trespass. Equally, since the act of taking fingerprints generally requires the cooperation of, or use of force on, the subject and would otherwise amount to an assault, statutory powers were enacted to enable the police to take fingerprints. Both involve physically intrusive acts. By contrast, the use of AFR Locate to obtain biometric information is very different. No physical entry, contact or force is necessary when using AFR Locate to obtain biometric data. It simply involves taking a photograph of someone's face and the use of algorithms to attempt to match it with photographic images of faces on a watchlist. The method is no more intrusive than the use of CCTV in the streets.^11^

The court also addressed the fact that the AFR Locate technology was new and not governed by specific legislation, finding that this did not preclude its use by SWP either:

In our view, there is a clear and sufficient legal framework governing whether, when and how AFR Locate may be used. What is important is to focus on the substance of the actions that use of AFR Locate entails, not simply that it involves a first-time deployment by SWP of an emerging technology. The fact that a technology is new does not mean that it is outside the scope of existing regulation, or that it is always necessary to create a bespoke legal framework for it. The legal framework within which AFR Locate operates comprises three elements or layers (in addition to the common law), namely: (a) primary legislation; (b) secondary legislative instruments in the form of codes of practice issued under primary legislation; and (c) SWP's own local policies. Each element provides legally enforceable standards. When these elements are considered collectively against the backdrop of the common law, the use of AFR Locate by SWP is sufficiently foreseeable and accessible for the purpose of the “in accordance with the law” standard.^12^

In addition to judicial review by the courts, an independent statutory commissioner has been appointed to respond to concerns relating to consent, retention and use of biometric information in the United Kingdom, the Commissioner for the Retention and Use of Biometric Material.^13^ The mandate of the Commissioner is to regulate the use of biometric information and provide a degree of protection from disproportionate law enforcement action.^14^ They have statutory powers that include oversight of the retention of biometric information by deciding on applications made by police to retain biometric information, as well as reporting to the Home Secretary about these functions or other matters considered appropriate. Significantly, the Commissioner’s powers do not currently extend to biometric information other than DNA or fingerprints (OBC 2020); however, the House of Commons Science and Technology Committee has recommended that the statutory responsibilities of the Biometrics Commissioner ‘be extended to cover, at a minimum, the police use and retention of facial images’ (HCSTC 2015; 34).

## Ethical principles

The expanding use of biometric facial recognition raises a number of pressing ethical concerns for liberal democracies that need to be considered. The concerns relate especially to the potential conflicts between security, on the one hand; and individual privacy and autonomy, and democratic accountability, on the other. Security and community safety are fundamental values in liberal democracies, as in other polities, including many authoritarian ones. However, liberal democracies are also committed to individual privacy and autonomy, democracy, and, therefore, democratic accountability. Accordingly, the latter fundamental ethical principles must continue to be valued in liberal democracies such as Australia, the United Kingdom and the United States, notwithstanding the benefits to security and community safety that biometric facial recognition can provide (Miller and Bossomaier 2021). While debates will continue between proponents of security, on the one hand, and defenders of privacy, on the other, there is often a lack of clarity in relation to the values or principles allegedly in conflict.

### Privacy

The notion of privacy has proven difficult to adequately explicate. Nevertheless, there are a number of general points that can be made. First, privacy is a right that people have in relation to other persons, the state and organisations with respect to: (a) the possession of information (including facial images) about themselves by other persons and by organisations, e.g. personal information and images stored in biometric databases, or; (b) the observation/perceiving of themselves—including of their movements, relationships and so on—by other persons, e.g. via surveillance systems including tracking systems that rely on biometric facial images (Kleinig et al. 2011). Biometric facial recognition is obviously implicated in both informational and observational concerns.

Second, the right to privacy is closely related to the more fundamental moral value of autonomy. Roughly speaking, the notion of privacy delimits an informational and observational ‘space’ i.e. the private sphere. However, the right to autonomy consists of a right to decide what to think and do and, of relevance here, the right to control the private sphere and, therefore, to decide *who to exclude and who not to exclude* from it (Kleinig et al. 2011). So the right to privacy consists of the right to exclude organisations and other individuals (the right to autonomy) both from personal information and facial images, and from observation and monitoring (the private sphere). Naturally, the right to privacy is not absolute; it can be overridden. Moreover, its precise boundaries are unclear; a person does not have a right not to be observed in a public space but, arguably, has a right not to be photographed in a public space (let alone have an image of their face widely circulated on the internet), albeit this right not to be photographed and have one’s image circulated can be overridden under certain circumstances. For instance, this right might be overridden if the public space in question is under surveillance by CCTV to detect and deter crime, and if the resulting images are only made available to police—and then only for the purpose of identifying persons who have committed a crime in that area. What of persons who are present in the public space in question and recorded on CCTV, but who have committed a serious crime, such as terrorism, elsewhere, or at least are suspected of having committed a serious crime^15^ elsewhere and are, therefore, on a watch list? Presumably, it is morally acceptable to utilise CCTV footage to identify these persons as well. If so, then it seems morally acceptable to utilize biometric facial recognition technology to match images of persons recorded on CCTV with those of persons on a watch list of those who have committed, for instance, terrorist actions, or are suspected of having done so, as the SWP were arguably seeking to do in the *Bridges* case.

Third, a degree of privacy is necessary simply for people to pursue their personal projects, whatever those projects might be. For one thing, reflection is necessary for planning, and reflection requires a degree of freedom from the distracting intrusions, including intrusive surveillance, of others (Kleinig et al. 2011). For another, knowledge of someone else’s plans can lead to those plans being thwarted (e.g. if one’s political rivals can track one’s movements and interactions then they can come to know one’s plans in advance of their implementation), or otherwise compromised, (e.g. if who citizens vote for is not protected by a secret ballot, including a prohibition on cameras in private voting booths, then democracy can be compromised).

We have so far considered the rights of a *single* individual; however, it is important to consider the implications of the infringement, indeed violation, of the privacy and autonomy rights of the whole citizenry by the state (and/or other powerful institutional actors, such as corporations). Such violations on a large scale can lead to a power imbalance between the state and the citizenry and, thereby, undermine liberal democracy itself (Miller and Walsh 2016). The surveillance system imposed on the Uighurs in China, incorporating biometric facial recognition technology, graphically illustrates the risks attached to large-scale violations of privacy and related autonomy rights.

Accordingly, while it is morally acceptable to collect biometric facial images for necessary circumscribed purposes, such as passports for border control purposes and drivers’ licences for safety purposes, it is not acceptable to collect them to establish vast surveillance states as China has done, and exploit them to discriminate on the basis of ethnicity. However, images in passports and driving licences are, and arguably ought to be, available for *wider* law enforcement purposes, e.g. to assist in tracking the movements of persons suspected of serious crimes unrelated to border control or safety on the roads. The issue that now arises is the determination of the point on the spectrum at which privacy and security considerations are appropriately balanced.

Privacy can reasonably be overridden by security considerations under some circumstances, such as when lives are at risk. After all, the right to life is, in general, a weightier moral right than the right to privacy (Miller and Walsh 2016). Thus utilising facial recognition technology to investigate a serious crime such as a murder or track down a suspected terrorist, if conducted under warrant, is surely ethically justified. On the other hand, intrusive surveillance of a suspected petty thief might not be justified. Moreover, given the importance of, so to speak, the aggregate privacy/autonomy of the citizenry, threats to life on a small scale might not be of sufficient weight to justify substantial infringements of privacy/autonomy, e.g. a low level terrorist threat might not justify citizen-wide biometric facial recognition database. Further, regulation, and associated accountability mechanisms need to be in place to ensure that, for instance, a database of biometric facial images created for a legitimate purpose, e.g. a repository of passport photos, can be accessed by border security and law enforcement officers to enable them to prevent and detect serious crimes, such as murder, but not used to identify protesters at a political rally.

We have argued that privacy rights, including in respect of biometric facial images, are important, in part because of their close relation to autonomy, and although they can be overridden under some circumstances, notably by law enforcement investigations of serious crimes, there is obviously a point where infringements of privacy rights is excessive and unwarranted. A national biometric facial recognition database for use in relation to serious crimes, and subject to appropriate accountability mechanisms may be acceptable, but utilising billions of images from social media accounts (e.g. in the way that Clearview AI’s technology does) to detect and deter minor offences, let alone establishing a surveillance state (e.g. to the extent that has been achieved in China), is clearly unacceptable. Let us now turn directly to security.

### Security and public safety

Security can refer to, for example, national security (such as harm to public from a terrorist attack), community security (such as in the face of disruptions to law and order) and organisational security (such as breaches of confidentiality and other forms of misconduct and criminality). At other times it is used to refer to personal physical security. Physical security in this sense is security in the face of threats to one’s life, freedom or personal property—the latter being goods to which one has a human right. Violations or breaches of physical security obviously include murder, rape, assault and torture (Miller and Bossomaier 2021). Biometric facial recognition systems could assist in multiple ways to enhance security in each of these senses. Thus a biometric facial recognition system could help to prevent fraud by better establishing identity (e.g. identify people using falsified drivers licences) and facial recognition data would be likely to help to investigate serious crimes against persons, such as murder and assault (e.g. identifying unknown suspects via CCTV footage).

Arguably, security should be distinguished from safety, although the two concepts are related and the distinction somewhat blurred. We tend to speak of safety in the context of wildfires, floods, pandemics and the like, in which the harm to be avoided is not intended harm. By contrast, the term ‘security’ typically implies that the threatened harm is intended. At any rate, it is useful to at least maintain a distinction between intended and unintended harms and, in relation to unintended harms, between foreseen, unforeseen and unforeseeable harms. For instance, someone who is unknowingly carrying the COVID-19 virus because they are asymptomatic, is a danger to others but, nevertheless, might not be culpable (if, for instance, they had taken reasonable measures to avoid being infected, had an intention to test for infection if symptoms were to arise and, if infected, would take all possible measures not to infect others). While biometric facial recognition systems can make an important contribution to security, their utility in relation to safety is less obvious, albeit they could assist in relation to finding missing persons or ensuring unauthorised persons do not unintentionally access dangerous sites (Miller and Smith 2021).

A number of potential ethical problems arise from the expanding use of biometric facial recognition for security purposes, especially in the context of interlinkage with non-biometric databases, data analytics and artificial intelligence. First, the security contexts in which their use is to be permitted might become both very wide and continuing, e.g. the counter-terrorism (‘emergency’) security context becomes the ‘war’ (without end) against terrorism; which becomes the war (without end) against serious crime; which becomes the ‘war’ (without end) against crime in general (Miller and Gordon 2014).

Second, data, including surveillance data, originally and justifiably gathered for one purpose, e.g. taxation or combating a pandemic, is interlinked with data gathered for another purpose, e.g. crime prevention, without appropriate justification. The way metadata use has expanded from initially being used by only a few agencies to now being used quite widely by governments in many western countries, is an example of function creep and illustrates the potential problems that might arise with the introduction of biometric facial recognition systems (Mann and Smith 2017).

Third, various general principles taken to be constitutive of liberal democracy are gradually undermined, such as the principle that an individual has a right to freedom from criminal investigation or unreasonable monitoring, absent prior evidence of violation by that individual of its laws. In a liberal democratic state, it is generally accepted that the state has no right to seek evidence of wrongdoing on the part of a particular citizen or to engage in selective monitoring of that citizen, if the actions of the citizen in question have not otherwise reasonably raised suspicion of unlawful behaviour and if the citizen has not had a pattern of unlawful past behaviour that justify monitoring. Moreover, in a liberal democratic state, it is also generally accepted that there is a presumption against the state monitoring the citizenry. This presumption can be overridden for specific purposes but only if the monitoring in question is not disproportionate, is necessary or otherwise adequately justified and kept to a minimum, and is subject to appropriate accountability mechanisms. Arguably, the use of CCTV cameras in crime hot-spots could meet these criteria if certain conditions were met, e.g. police access to footage was granted only if a crime was committed or if the movements of a person reasonably suspected of a crime needed to be tracked. However, these various principles are potentially undermined by certain kinds of offender profiling and, specifically, ones in which there is no specific (actual or reasonably suspected) past, imminent or planned crime being investigated. Biometric facial recognition could be used to facilitate, for instance, a process of offender profiling, risk assessment and subsequent monitoring of people who as a result of fitting these profiles are considered at risk of committing crimes, notwithstanding that the only offences that the individuals in question had committed was to fit these profiles.

## Conclusion

We have described the expanding use of biometric facial recognition for security and public safety purposes and elaborated on current applications and legal developments in Australia, the United States and the United Kingdom. In light of these applications and developments, we have outlined the relevant ethical principles and identified a number of actual or potential problems that arise in relation to this rapidly developing form of information technology.

We conclude with a number of general points that ought to guide policy in this area. First, privacy in relation to personal data, such as facial images, consists in large part in the right to control the access to, and use of, that data. Moreover, security consists in large part in individual rights, notably the right to life, as well as to institutional goods, such as law and order. Biometric facial recognition technology gives rise to security concerns, such as the possibility of identity theft by a sophisticated malevolent actor, even as they resolve old privacy and confidentiality concerns, such as by reducing unauthorised access to private information and thereby strengthening privacy protection. In short, the problems in this area cannot be framed in terms of a simple weighing of, let alone trade-off between, individual privacy rights versus the community’s interest in security.

Second, the establishment of comprehensive, integrated biometric facial recognition databases and systems by governments (and now the private sector), and the utilisation of this data to identify and track citizens, (e.g. via live CCTV feeds) has the potential to create a power imbalance between governments and citizens, and risks undermining important principles taken to be constitutive of the liberal democratic state, such as privacy.

Third, the expanding use of biometric facial recognition databases and systems has to be clearly and demonstrably justified in terms of efficiency and effectiveness in the service of *specific* security and/or safety purpose, rather than by general appeals to community security or safety*.*

Finally, in so far as the use of facial recognition and other biometric identification systems can be justified for specific security (and safety) purposes and, therefore, privacy and other concerns mitigated, it is, nevertheless, imperative that their use be subject to accountability mechanisms to guard against misuse. Citizens should be well informed about biometric facial recognition systems and should have consented to the use of these systems for the specific, justified purposes in question. Their use should be publicly debated, backed by legislation, and their operation subject to judicial review.

## Footnotes

1 For example, the Australian National University data breach, made public in 2019, involved data relating to 200,000 people extending over a 19 years period, believed to have been perpetrated by a ‘sophisticated state actor’.

2 The regulations were made pursuant to the *Road Transport (Driver Licensing) Act 1998* (NSW), which was later repealed by Schedule 1 of the *Road Transport Legislation (Repeal and Amendment) Act 2013* (NSW).

3 The *Road Transport Legislation Amendment (Release of Stored Photographs) Regulation 2015* (NSW). Section 57(1)(k) of the *Road Transport Act 2013* (NSW) outlines the circumstances in which these photographs can be released, including to the NSW Police Force.

4 Defined, for example, under the *Crime Commission Act 2012* (NSW) s (4)(1) as ‘any circumstances implying, or any allegations, that a relevant offence may have been, or may be being, or may in the future be, committed’. The Act defines ‘relevant offence’ in s 5(1) as ‘an offence that is punishable by imprisonment for life or for a term of 3 or more years’.

5 A ‘terrorist act’ is defined under the *Terrorism (Police Powers) Act 2002* (NSW) s 3(1) as when an ‘action is done with the intention of advancing a political, religious or ideological cause’ and with the intention of ‘coercing, or influencing by intimidation, the government of the Commonwealth or a State, Territory or foreign country’ or ‘intimidating the public or a section of the public’.

6 Statement of Claim, *State of Vermont v Clearview AI, Vermont Superior Court*, 10 March 2020, 8.

7 Haeggquist & Eck, LLP, *Sean Burke and James Pomerene, Individually and on Behalf of All Others Similarly Situated, Plaintiffs v. Clearview AI, Inc., a Delaware Corporation; Hoan Ton-That, an Individual; Richard Schwartz, an Individual; and Does 1 through 10, inclusive, Defendants*, United States District Court Southern District of California. Class Action Complaint Demand for Jury Trial. Case Number: 20CV0370 BAS MSB, 5–8.

8 [2019] EWHC 2341.

9 AFR (Automated Facial Recognition).

10 European Convention on Human Rights Article 8—Right to respect for private and family life. 1. Everyone has the right to respect for his private and family life, his home and his correspondence. 2. There shall be no interference by a public authority with the exercise of this right except such as is in accordance with the law and is necessary in a democratic society in the interests of national security, public safety or the economic well-being of the country, for the prevention of disorder or crime, for the protection of health or morals, or for the protection of the rights and freedoms of others.

11 Above n. 8, 75.

12 Above, n. 8, 84.

13 The UK Biometrics Commissioner was established under the *Protection of Freedoms Act 2012* (UK) in response to the judgement in the *S and* *Marper* *v United Kingdom* [2008] ECHR 1581 case in the European Court of Human Rights in 2008.

14 *Protection of Freedoms Act 2012* (UK) c 9, s 20.

15 Drawing on the legislative example (s 5(1) *Crime Commission Act 2012* (NSW)) stated above in note 4, we will define a serious crime as an offence punishable by imprisonment for life, or for a term of 3 or more years.
